# Early warning signs and comorbidities of attention deficit hyperactivity disorder in children in Western China: a multicenter, cross-sectional study

**DOI:** 10.1186/s12889-025-26014-8

**Published:** 2025-12-17

**Authors:** Chunbo Wang, Zhijuan Li, Jiangling Su, Mo Wang, Huan Yang, Gaofu Zhang

**Affiliations:** 1 The First Affiliated Hospital of Chongqing Medical University, Chongqing, 400016 China; 2 Department of Nephrology, Xi’an Children’s Hospital, The Affiliated Children’s Hospital of Xi’an Jiaotong University, Xi’an, 710003 China; 3 Department of Nephrology and Hematology, Yuxi Children’s Hospital, Yunnan, 653100 China; 4 Department of Nephrology，National Clinical Research Center for Child Health and Disorders, Ministry of Education Key Laboratory of Child Development and Disorders, International Science and Technology Cooperation base of Child development and Critical Disorders, Chongqing Key Laboratory of Pediatric Metabolism and Inflammatory Diseases, Children’s Hospital of Chongqing Medical University, Chongqing, 400014 China; 5 Department of Endoscope, National Clinical Research Center for Child Health and Disorders, Ministry of Education Key Laboratory of Child Development and Disorders, International Science and Technology Cooperation base of Child development and Critical Disorders, Chongqing Key Laboratory of Pediatric Metabolism and Inflammatory Diseases, Children’s Hospital of Chongqing Medical University, Chongqing, 400014 China

**Keywords:** Attention-Deficit/Hyperactivity disorder, Comorbidity, Rural china, Family environment, Sleep disorders, Problem behaviors

## Abstract

**Background:**

This study aimed to investigate the prevalence of attention-deficit/hyperactivity disorder (ADHD) and its association with common comorbidities among children in rural western China, to improve early recognition of ADHD in children by identifying early signs.

**Methods:**

A cross-sectional study was conducted with children aged 6–18 in rural schools across three provinces in western China. ADHD symptoms were assessed through caregiver-reported questionnaires utilizing the Swanson, Nolan, and Pelham Questionnaire-IV (SNAP-IV), without the involvement of clinical diagnosis. Propensity score matching and conditional logistic regression were used to analyze associations between ADHD risk and comorbidities while controlling for potential confounders.

**Results:**

2.07% of children were at risk for ADHD, with only 3.25% of them receiving a diagnosis or treatment. The risk of ADHD was significantly associated with the presence of family disharmony, sleep disorders, behavioral abnormalities, and parental anxiety and depression. After matching, the strongest associations were found with abnormal behavior (OR = 6.467, *P* < 0.001) and daytime sleepiness (OR = 1.128, *P* < 0.001). Age-stratified analysis revealed that family conflict, daytime sleepiness, abnormal behavior, and parental anxiety symptoms were consistently associated with higher ADHD risk in children aged 6–15 years.

**Conclusions:**

In this cross-sectional study, the low diagnosis and treatment rates for ADHD in rural western China highlight significant gaps in recognition and intervention. Children at risk for ADHD was associated with various comorbidities, particularly behavioral abnormalities and daytime sleepiness, suggesting that screening for ADHD should be incorporated into assessments of children presenting with these comorbidities, especially in regions with low ADHD awareness.

**Supplementary Information:**

The online version contains supplementary material available at 10.1186/s12889-025-26014-8.

## Background

Attention-deficit/hyperactivity disorder (ADHD) is a prevalent, chronic neurodevelopmental disorder, characterized by pervasive and impairing symptoms of inattention, hyperactivity, and impulsivity [1, 2]. Globally, about 5% of children and adolescents [3] and 2.5% of adults are affected [4]. In China, meta-analyses estimate ADHD’s prevalence among children and adolescents at approximately 6.3%, with geographic, socioeconomic, and methodological factors contributing to variability [5].

ADHD is frequently comorbid with other psychiatric and physical disorders, creating a substantial burden for the individual, their family, and the community [6]. Children with ADHD are more often rejected by their peers [7] and are at risk of negative academic outcomes [8]. Emotional dysregulation, including aggressive behavior, emotional lability, and poor frustration tolerance, is frequently observed [9, 10]. Furthermore, children with ADHD frequently have sleep disturbances and daytime sleepiness, which represent clinically important features that might be therapeutically targeted [11, 12].

Despite substantial evidence of ADHD’s impact and the consensus that health professionals should identify and treat the disorder [13, 14], significant gaps remain in rural and underdeveloped regions. ADHD is a clinical diagnosis requiring a comprehensive evaluation [15], which is often unavailable in low-resource settings [16]. Rural western China is characterized by socioeconomic disadvantage, limited access to mental health services, and lower parental awareness of ADHD [17]. Our previous study found that in this region, about 2% of children are at risk of ADHD, and only a small fraction (2.9%) had been diagnosed and treated [18]. This underscores an urgent public health need to identify pragmatic, early warning signs that can be recognized by parents and non-specialist physicians to facilitate earlier referral and intervention. Building on previous research, we expanded the scope of the study to further explore the relationship between ADHD risk and its comorbidities through a propensity score matching (PSM) analysis. The aim of this study is to improve early recognition of ADHD in children by identifying easily observable early signs and comorbidities, thereby enhancing the ability of parents and non-specialist physicians in rural western China to recognize ADHD early, facilitating timely intervention and comprehensive management.

## Methods

### Study setting and participants

A cross-sectional survey was carried out from January to December 2023. One prefecture-level city was randomly selected from the rural-dominated regions of each of the three provinces/municipality (Chongqing, Shaanxi, and Yunnan). These regions are representative of impoverished and relatively backward areas in western China. The target sample size was calculated based on an estimated ADHD prevalence of 6% in China, a margin of error of 0.5%, a confidence level of 95%, and a design effect of 2.0 for cluster sampling, yielding a target of approximately 36,000 students. Accounting for an estimated 75% response rate, we aimed to distribute 27,000–28,000 questionnaires across the three regions, with the final number per city proportional to the size of the school-age population in each selected rural area.

We used cluster sampling to randomly select twelve primary schools, ten junior high schools, and six senior high schools from a list covering all educational levels. Students aged 6 to 18 years were recruited, resulting in 27,508 returned questionnaires, with a response rate of approximately 75%. Exclusion criteria of the study included (1) children had an intellectual disability or a confirmed organic disease; (2) children had a history of surgery for the spine, spinal cord. Children with a history of surgery for the spine or spinal cord were excluded because such procedures and their underlying conditions can lead to neurological sequelae (e.g., sensory, motor, or cognitive deficits) that may mimic or confound the symptoms of ADHD, thus ensuring a more homogeneous sample for studying idiopathic ADHD.

### Procedure

The study was approved by the Institutional Review Board of Children’s Hospital of Chongqing Medical University (No.2024-73) and was performed in accordance with the Declaration of Helsinki. An anonymous online questionnaire was distributed to the students’ primary caregivers by teachers and completed by them. Informed consent to participate was obtained from the children’s legal guardians after explaining the study’s objectives and risks, and guardians had the right to refuse participation.

To ensure data quality, several measures were implemented. First, prior to data collection, primary caregivers participated in an online training session conducted by a specialized child psychological health physician. This session provided detailed guidance on how to understand and complete each scale, particularly the SNAP-IV, with opportunities for Q&A. Second, class teachers and school health workers were trained to assist caregivers and liaise with the research team for any queries. Third, the online questionnaire system was programmed with validation checks: it mandated the completion of all required items and monitored completion time; questionnaires submitted too quickly (e.g., under a predetermined minimum time) or with missing mandatory fields could not be submitted, prompting a review.

All survey data were consistently entered by trained staff to ensure standardized data management. Specialized personnel uniformly entered all survey data, ensuring confidentiality. The study was funded as a Chongqing Medical Scientific Research Project.

### Measures

The entire questionnaire consists of seven sections. Collected demographic data covered gender, age, height, weight, Body Mass Index (BMI), residential area, annual family income, the identity of primary caregiver, primary caregiver’s education level, parents’ marital status, number of children, pregnancy and childbirth history (including parental depression during pregnancy, which was assessed retrospectively via a single-item question), child’s academic performance, interpersonal relationships at school, and other factors. The complete demographic questionnaire is available in Additional file 1.

#### The Swanson, Nolan, Pelham-IV scale (SNAP-IV)

The SNAP-IV parent-report scale was used to assess ADHD symptoms. It contains 18 items, with nine assessing inattention and nine assessing hyperactivity/impulsivity, each rated on a 4-point scale (0 = never, 3 = very often). Consistent with established criteria [2], a child was considered to have clinically relevant symptoms if six or more items in either domain were rated as “often” or “very often”. Students meeting this threshold were classified as being at risk of ADHD. Those meeting criteria for only the inattention domain were classified as the inattentive subtype, those for only the hyperactivity/impulsivity domain as the hyperactive/impulsive subtype, and those for both domains as the combined subtype [19]. In this study, ADHD classification was strictly based on caregiver-reported SNAP-IV assessments, without teacher input or clinical confirmation. The Cronbach’s α in our study was 0.930.

#### Children’s sleep habits questionnaire (CSHQ)

Sleep behaviors were measured using the Children’s Sleep Habits Questionnaire (CSHQ), a parent-report instrument with 33 items grouped into eight subscales, including bedtime resistance, sleep onset delay, sleep duration, night wakings, parasomnias, sleep-disordered breathing, daytime sleepiness, and sleep anxiety. Items are rated on a 3-point frequency scale (“rarely,” “sometimes,” “usually”), and higher scores indicate more sleep difficulties. A total score ≥ 41 was used to indicate poor sleep quality, consistent with established cut-off values [20].

#### Rutter children’s behavior questionnaire (RCBQ)

Behavioral problems were assessed using the parent-report version of the Rutter Children’s Behavior Questionnaire (RCBQ), which screens for emotional difficulties, conduct problems, and hyperactivity. Items are rated on a 3-point scale (“does not apply,” “applies somewhat,” “certainly applies”), and higher scores reflect greater behavioral difficulties. Scores in the abnormal range (a total score ≥ 13) were classified as abnormal behavior [21].

#### Zung Self-Rating anxiety scale (SAS)

Caregivers’ anxiety symptoms were measured using the Zung Self-Rating Anxiety Scale (SAS). The SAS contains 20 items covering affective, cognitive, and somatic aspects of anxiety, rated on a 4-point scale. Total raw scores are multiplied by 1.25 to yield a standardized score (range: 25–100). A cutoff of 45 has been suggested for identifying clinically significant anxiety in Chinese samples [22, 23].

#### Zung Self-Rating depression scale (SDS)

Caregivers’ depressive symptoms were assessed using the Zung Self-Rating Depression Scale (SDS), which consists of 20 items rated on a 4-point scale. Standardized scores are obtained by multiplying raw scores by 1.25, with scores ≥ 50 indicating possible depressive symptoms [24].

#### Family environment scale (FES)

Family environment was assessed using an excerpt from the Family Environment Scale (FES), a widely used measure of family social environment with demonstrated reliability and validity. The FES includes subscales assessing family cohesion and family conflict, each comprising 10 items. Items are rated on a binary scale (“true” or “false”), with higher cohesion scores reflecting stronger emotional bonding and higher conflict scores reflecting more frequent interpersonal discord within the family [25].

### Statistical analyses

All statistical analyses were performed using the R software (version 4.2.2). Wilcoxon rank sum tests were used for non-parametric data, and Chi-square tests (or Fisher’s exact tests where appropriate) were used for proportions. Before conducting propensity score matching, we excluded students who were reported to be currently receiving pharmacological treatment for ADHD, to avoid potential confounding effects of medication on the assessment of comorbidities. To address potential confounding in this observational design, we used propensity score matching (PSM), which allows us to create a comparison group of children without ADHD risk who are similar in measured baseline characteristics to those at risk, thereby mimicking some features of randomized designs [26]. This approach provides more balanced groups and reduces bias from observed covariates compared to conventional regression adjustment alone. Propensity scores were estimated using all baseline characteristics with *P* < 0.1 from univariate analyses. A 1:4 PSM was performed using greedy nearest-neighbor matching with a caliper of 0.02 (R package MatchIt), matching each child at risk for ADHD with four children without ADHD risk.

After matching, univariate conditional logistic regression was performed to examine the independent association of each comorbidity variable with ADHD risk (Table 2). Variables with *P* < 0.1 in these univariate analyses were then included in a multivariate conditional logistic regression model to identify independent associations while controlling for other variables in the model (Table 3). Multicollinearity was assessed using Variance Inflation Factors (VIFs); all variables included had VIFs < 5, indicating no substantial multicollinearity. Finally, age-stratified subgroup analyses were conducted for variables significant in the multivariate model. The age groups were defined as: 6–9 years, 10–12 years, 13–15 years, and 16–18 years.

It should be noted that while PSM helped balance individual-level covariates, the matched analyses did not account for the potential intra-cluster correlation arising from the school-based sampling design. All P-values were two-sided, with *P* < 0.05 indicating statistical significance.

## Results

### Prevalence of ADHD risk and comorbidities

A total of 27,508 questionnaires were collected, of which 26,726 were valid after quality control. Among these, 554 children (2.07%) were identified as being at risk for ADHD according to SNAP-IV scores. Specifically, 281 (50.72%) were of the inattentive type, 63 (11.37%) of the hyperactive/impulsive type, and 210 (37.91%) of the combined type.

Strikingly, only 18 (3.25%) of these children at risk had ever received a clinical diagnosis and treatment, underscoring the extremely low treatment rate. Compared with their peers, children at risk for ADHD showed markedly higher frequencies of comorbidities: 22.02% for nocturnal enuresis, 23.10% for fecal incontinence, and 82.67% for poor sleep quality. In addition, 72.56% of them exhibited behavioral abnormalities. Among their families, 31.40% of parents reported anxiety symptoms, and 61.19% experienced depressive symptoms.

### Demographics of children with and without ADHD risk

Table 1 presents the demographic comparison between children with and without ADHD risk. The prevalence of ADHD risk was significantly higher among boys (66.06%, *P* < 0.001), those of shorter stature (*P* < 0.001), and children from divorced or remarried families (*P* < 0.001). Differences across age groups were also significant (*P* = 0.010). No significant group differences were observed in body weight, BMI, caregiver’s education level, number of children in the household, or annual family income. The complete dataset for Table 1 is available in the Additional file 2, Table S1.

In terms of prenatal and perinatal factors, passive smoking during pregnancy (*P* < 0.001) and parental depression during pregnancy (*P* < 0.001) were both significantly associated with an increased ADHD risk, while birth weight (*P* = 0.009) was lower among children at risk. Gestational weeks and alcohol consumption did not differ between groups.

**Table 1:** Selected baseline characteristics of participants with and without ADHD risk

*N*	Overall	Non-ADHD risk	ADHD risk	*P*-value
	26,726	26,172	554	
Sex				< 0.001^2^
Male	13,445 (50.31%)	13,079 (49.97%)	366 (66.06%)	
Female	13,281 (49.69%)	13,093 (50.03%)	188 (33.94%)	
Age groups				0.010^2^
6–9	10,258 (38.38%)	10,018 (38.28%)	240 (43.32%)	
10–12	8,974 (33.58%)	8,800 (33.62%)	174 (31.41%)	
13–15	5,303 (19.84%)	5,191 (19.83%)	112 (20.22%)	
16–18	2,191 (8.20%)	2,163 (8.26%)	28 (5.05%)	
Height	145 (130, 157)	145 (130, 157)	140 (130, 153)	< 0.001^3^
City	12,473 (46.67%)	12,215 (46.67%)	258 (46.57%)	
City-country fringe	6,155 (23.03%)	6,016 (22.99%)	139 (25.09%)	
Country	8,098 (30.30%)	7,941 (30.34%)	157 (28.34%)	
Primary caregiver				< 0.001^2^
Mother	18,425 (68.94%)	18,077 (69.07%)	348 (62.82%)	
Father	2,408 (9.01%)	2,353 (8.99%)	55 (9.93%)	
Paternal grandparents	3,601 (13.47%)	3,499 (13.37%)	102 (18.41%)	
Maternal grandparents	1,356 (5.07%)	1,328 (5.07%)	28 (5.05%)	
Nanny	21 (0.08%)	19 (0.07%)	2 (0.36%)	
Others	915 (3.42%)	896 (3.42%)	19 (3.43%)	
Marital status of parents				< 0.001^2^
Non-special	22,355 (83.65%)	21,931 (83.80%)	424 (76.53%)	
Divorced	2,830 (10.59%)	2,752 (10.52%)	78 (14.08%)	
Reconstituted	1,218 (4.56%)	1,174 (4.49%)	44 (7.94%)	
Widowed	323 (1.21%)	315 (1.20%)	8 (1.44%)	
Birth weight				0.009^4^
˂1 kg	115 (0.43%)	109 (0.42%)	6 (1.08%)	
1–1.5 kg	1,249 (4.67%)	1,210 (4.62%)	39 (7.04%)	
1.5–2.5 kg	4,118 (15.41%)	4,041 (15.44%)	77 (13.90%)	
2.5–4 kg	20,273 (75.85%)	19,865 (75.90%)	408 (73.65%)	
˃4 kg	971 (3.63%)	947 (3.62%)	24 (4.33%)	
Passive smoking in pregnancy	3,695 (13.83%)	3,549 (13.56%)	146 (26.35%)	< 0.001^2^
Depressed in pregnancy				
mom	1,957 (7.32%)	1,832 (7.00%)	125 (22.56%)	< 0.001^2^
dad	371 (1.39%)	350 (1.34%)	21 (3.79%)	< 0.001^2^

^1^n (%); Median (IQR), ^2^Pearson's Chi-squared test, ^3^Wilcoxon rank sum test, ^4^Fisher's exact test

### Association of ADHD risk with comorbidities

**Fig. 1:**
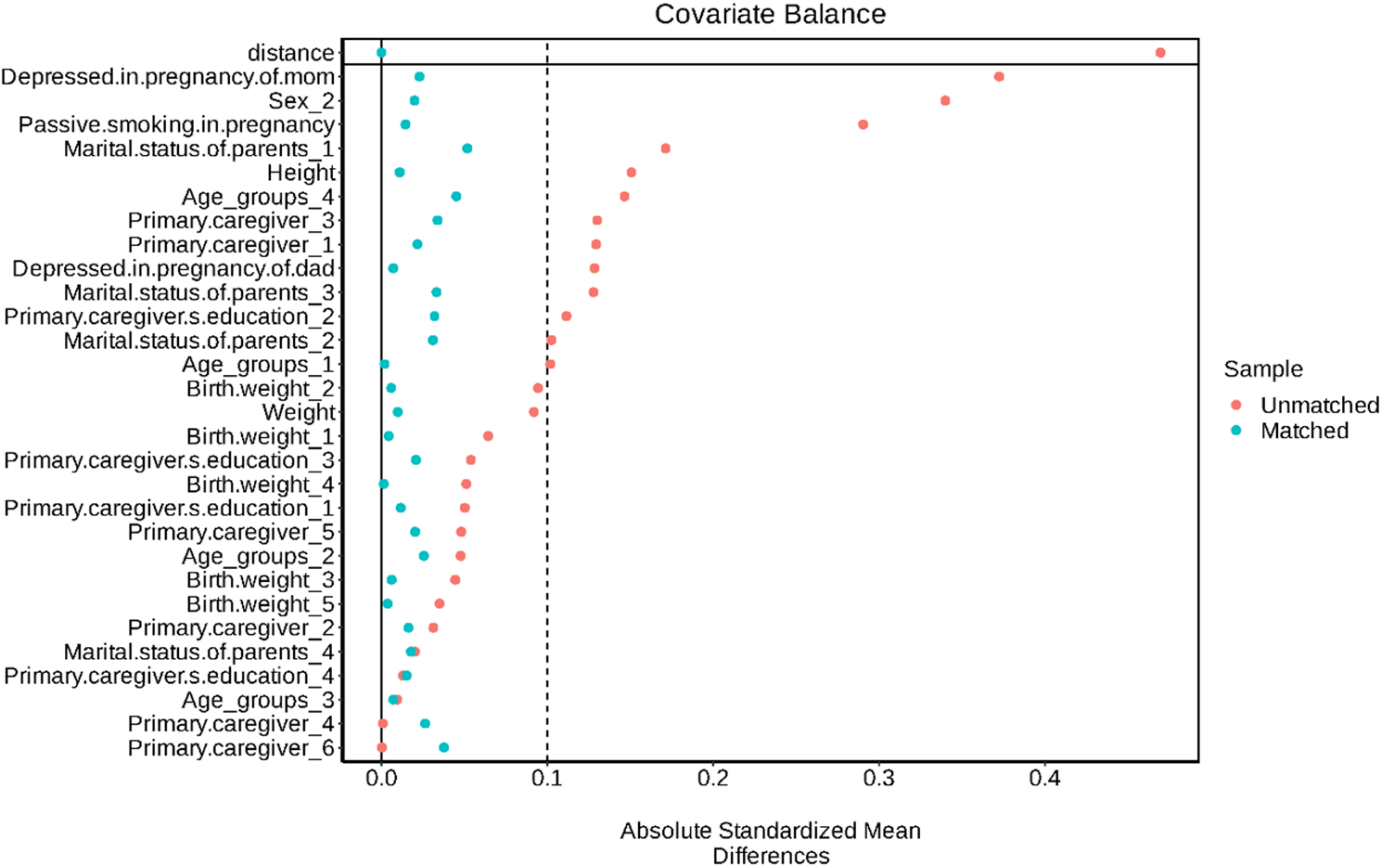
Covariate balance: before vs. after propensity score matching

After excluding students under potential pharmacologic interference, 547 children at risk for ADHD were matched with 2,128 controls using 1:4 propensity score matching (PSM). The post-matching standardized mean differences (SMDs) were notably reduced, suggesting improved covariate balance and minimized confounding (Fig. 1).

To further explore the differences between groups, a series of conditional logistic regression analyses were performed (Table 2). The univariate results revealed that the risk of ADHD was significantly associated with multiple family and behavioral factors. Specifically, lower family cohesion (OR = 0.910, *P* < 0.001) and higher family conflict (OR = 1.138, *P* < 0.001) were both related to an increased ADHD risk. In addition, several dimensions of sleep problems—including bedtime resistance (OR = 1.137, *P* < 0.001), sleep onset delay (OR = 1.625, *P* < 0.001), shorter sleep duration (OR = 1.270, *P* < 0.001), sleep anxiety (OR = 1.146, *P* < 0.001), night wakings (OR = 1.132, *P* = 0.003), parasomnias (OR = 1.127, *P* < 0.001), sleep-disordered breathing (OR = 1.196, *P* < 0.001), and daytime sleepiness (OR = 1.264, *P* < 0.001)—showed significant positive associations with ADHD risk. Children with ADHD risk also demonstrated markedly higher odds of overall sleep disorder (OR = 3.872, *P* < 0.001), abnormal behavior (OR = 10.758, *P* < 0.001), parental depressive symptoms (OR = 2.117, *P* < 0.001), and parental anxiety symptoms (OR = 3.800, *P* < 0.001) than those without risk.

Prior to multivariate analysis, variance inflation factors (VIFs) were assessed to test for multicollinearity, and all included variables had VIF values < 5, indicating no evidence of multicollinearity (detailed values are presented in Additional file 2, Table S2). In the multivariate conditional logistic regression model (Table 3), ADHD risk remained independently associated with lower family cohesion (OR = 0.971, *P* = 0.019), higher family conflict (OR = 1.040, *P* = 0.007), night wakings (OR = 0.827, *P* = 0.019), daytime sleepiness (OR = 1.128, *P* < 0.001), abnormal behavior (OR = 6.467, *P* < 0.001), and parental anxiety symptoms (OR = 1.425, *P* = 0.028). Among these, the strongest associations were observed for daytime sleepiness and abnormal behavior.

**Table 2:** Analyses of the association of ADHD risk with comorbidities after propensity score matching

Continuous Variables	Non-ADHD risk (*n* = 26,172)	ADHD risk (*n* = 554)	OR (95% CI)	*P*-value
	Median (IQR)	Median (IQR)		
Cohesion	10.0 (5.0, 14.0)	4.0 (0.0, 9.0)	0.910 (0.896–0.924)	< 0.001
Conflict	-8.0 (-11.0, -4.0)	-3.0 (-7.0, -0.8)	1.138 (1.116–1.160)	< 0.001
Bedtime resistance	7.00 (6.00, 9.00)	8.00 (7.00, 11.00)	1.137 (1.097–1.177)	< 0.001
Sleep onset delay	1.00 (1.00, 2.00)	1.00 (1.00, 2.00)	1.625 (1.418–1.862)	< 0.001
Sleep duration	5.00 (3.00, 6.00)	6.00 (4.00, 7.00)	1.270 (1.195–1.349)	< 0.001
Sleep anxiety	4.00 (4.00, 6.00)	5.00 (4.00, 7.00)	1.146 (1.097–1.198)	< 0.001
Night wakings	3.00 (3.00, 3.00)	3.00 (3.00, 4.00)	1.132 (1.044–1.228)	0.003
Parasomnias	7.00 (7.00, 8.00)	8.00 (7.00, 10.00)	1.127 (1.086–1.170)	< 0.001
Sleep disordered breathing	3.00 (3.00, 3.00)	3.00 (3.00, 4.00)	1.196 (1.101–1.299)	< 0.001
Daytime sleepiness	12.00 (10.00, 14.00)	14.00 (12.00, 16.00)	1.264 (1.221–1.308)	< 0.001
Categorical Variables	n (%)	n (%)		
Sleep disorders				
No (Reference)	12,789 (48.87%)	96 (17.33%)	1.00	-
Yes	13,383 (51.13%)	458 (82.67%)	3.872 (3.038–4.935)	< 0.001
Abnormal behavior				
No (Reference)	22,146 (84.62%)	152 (27.44%)	1.00	-
Yes	4,026 (15.38%)	402 (72.56%)	10.758 (8.440-13.713)	< 0.001
Depression symptoms (SDS)				
No (Reference)	16,092 (61.49%)	215 (38.81%)	1.00	-
Yes	10,080 (38.51%)	339 (61.19%)	2.117 (1.744–2.568)	< 0.001
Anxiety symptoms (SAS)				
No (Reference)	24,092 (92.05%)	380 (68.59%)	1.00	-
Yes	2,080 (7.95%)	174 (31.41%)	3.800 (3.004–4.808)	< 0.001

Figure 1 presents the standardized mean differences (SMDs) before and after propensity.

**Table 3:** Multivariate analysis of the association of ADHD risk with comorbidities

	OR (95%CI)	*P* value
Cohesion	0.971 (0.947–0.995)	0.019
Conflict	1.040 (1.011–1.070)	0.007
Bedtime resistance	1.021 (0.945–1.103)	0.598
Sleep onset delay	1.091 (0.903–1.319)	0.366
Sleep duration	0.961 (0.952–1.065)	0.392
Sleep anxiety	1.032 (0.931–1.143)	0.448
Night wakings	0.827 (0.706–0.969)	0.019
Parasomnias	1.018 (0.858–1.206)	0.069
Sleep disordered breathing	1.018 (0.858–1.206)	0.841
Daytime sleepiness	1.128 (1.074–1.184)	< 0.001
Sleep disorders	1.132 (0.774–1.655)	0.524
Abnormal behavior	6.467 (4.964–8.425)	< 0.001
Depression symptoms (SDS)	0.845 (0.642–1.112)	0.228
Anxiety symptoms (SAS)	1.425 (1.038–1.957)	0.028

### Association of ADHD with selected comorbidities stratified by age

**Fig. 2:**
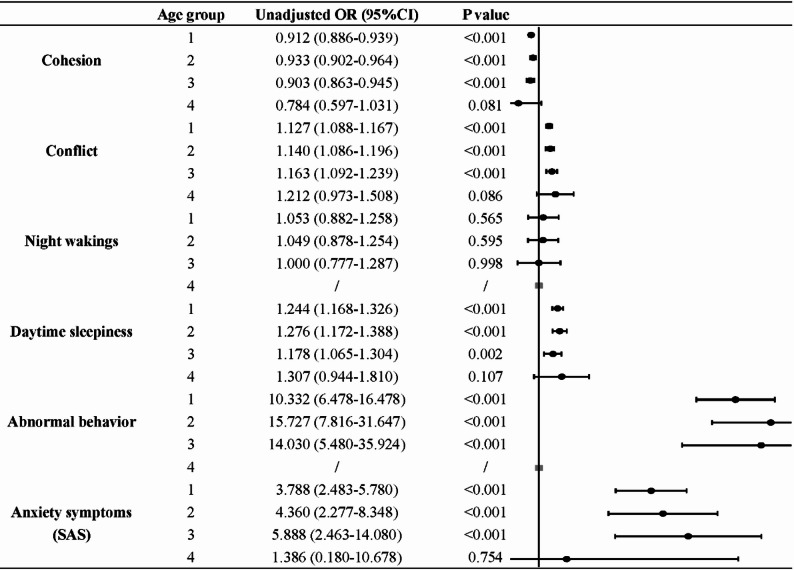
The association between ADHD risk and selected comorbidities stratified by age

Figure 2 shows the age-stratified conditional logistic regression results for comorbidities that were significant in multivariate analyses. Among adolescents aged 16–18 years, lower family cohesion, higher family conflict, night wakings, daytime sleepiness, abnormal behavior, and parental anxiety were not significantly related to ADHD risk, likely due to the smaller sample size in this subgroup.

In contrast, for children aged 6–15 years, ADHD risk was positively associated with reduced family cohesion, greater family conflict, daytime sleepiness, abnormal behavior, and parental anxiety symptoms, whereas night wakings were not significantly correlated at any age level. Moreover, the association between ADHD risk and both family conflict and parental anxiety demonstrated an approximately linear increase across the 6–15 age range.

## Discussion

This large cross-sectional study in rural western China identified 2.07% of children as being at risk for ADHD, with a strikingly low diagnosis and treatment rate of 3.25%. Our findings, utilizing propensity score matching to enhance comparability, reveal that behavioral abnormalities and daytime sleepiness are the strongest independent indicators of ADHD risk in this setting.

In our sample, we observed a very strong association between behavioral abnormalities and ADHD risk (OR = 6.467). This suggests that abnormal behavior patterns, as assessed by the RCBQ (including disruptive, oppositional, lying, aggressive, or stealing behaviors), may serve as highly accessible early warning indicators of ADHD, especially in rural settings where more subtle inattention symptoms might be overlooked. Research indicates that these behaviors are often associated with hyperactivity and impulsivity due to these psychological mechanism impairments [27]. Genome-wide association studies (GWAS) has made significant progress in identifying numerous genetic variants that are associated with ADHD and antisocial behavior (ASB), thereby providing valuable insights into the underlying genetic mechanisms of these complex phenotypes [28]. A recent study has confirmed the causal effect of ADHD on ASB and revealed potential causal mechanisms, using Mendelian randomization and enrichment analysis [29].

Another key finding was the robust association between daytime sleepiness and ADHD risk. The relationship is complex and likely bidirectional; ADHD may intrinsically cause sleep problems, while sleep issues may also mimic or exacerbate ADHD symptoms [30, 31]. This underscores the clinical importance of inquiring about sleep when assessing a child for potential ADHD, and vice versa. Significant associations were also found with family conflict and lower family cohesion, consistent with previous literature [32–34]. Family conflict can exacerbate symptoms, while family cohesion can serve as a protective factor, enhancing a child’s ability to cope with challenges [35]. Our age-stratified analysis provided valuable insights, indicating that the identified warning signs are particularly salient for children aged 6–15 years.

Our study has several limitations. First, data were collected through an online survey rather than in-person interviews, which may reduce response depth. Second, information on ADHD symptoms, sleep, behavior, and parental mental health was based exclusively on caregiver reports, without teacher input or clinical confirmation, increasing the risk of reporting bias and misclassification. Third, the assessment of parental depression during pregnancy was retrospective and non-standardized. Fourth, the cross-sectional design precludes causal inference. Finally, while PSM balanced observed confounders, it cannot account for unmeasured confounding, and the analyses did not adjust for the cluster sampling design.

Despite these limitations, our study highlights that in resource-limited settings like rural western China, easily observable signs such as significant behavioral problems and pronounced daytime sleepiness should trigger consideration of ADHD screening, facilitating earlier recognition and referral.

## Conclusions

In rural western China, 2.07% of children were identified as being at risk for ADHD, yet the treatment rate remained extremely low. Children at risk for ADHD exhibited markedly higher rates of comorbidities, particularly behavioral abnormalities and daytime sleepiness. These findings highlight the urgent need for improved early screening and awareness. Focusing on these practical, observable warning signs could empower parents and non-specialist healthcare workers in underserved regions to identify children who may benefit from further assessment for ADHD.

## Supplementary Information

Supplementary Material 1.

Supplementary Material 2.

## Data Availability

The data that support the findings of this study are available from Gaofu Zhang but restrictions apply to the availability of these data, which were used under license for the current study, and so are not publicly available. Data are however available from the authors upon reasonable request and with permission of Gaofu Zhang.

## Abbreviations

ADHD: Attention-deficit/hyperactivity disorder
PSM: propensity score matching
SNAP-IV: The Swanson, nolan, pelham-iv scale
BMI: Body Mass Index
FES: The Family environment scale
CSHQ: The Children’s sleep habits questionnaire
RCBQ: The parent-reported rutter children’s behavior questionnaire
SAS: The zung self-rating anxiety scale
SDS: The zung self-rating depression scale
