# Supplementary material for: Early warning signs and comorbidities of attention deficit hyperactivity disorder in children in Western China: a multicenter, cross-sectional study
Source: BMC Public Health. 2025 Dec 17;26:268. doi: 10.1186/s12889-025-26014-8 (PMC12822042; doi:10.1186/s12889-025-26014-8)
Supplement: Supplementary file 1 — Supplementary Material 1. [file 12889_2025_26014_MOESM1_ESM.docx]

**Table S1: Complete Baseline Characteristics of Participants with and without ADHD risk**

|  | **Overall** | **Non-ADHD risk** | **ADHD risk** | *P-value* |
| --- | --- | --- | --- | --- |
| N | **26,726** | **26,172** | **554** |  |
| **Sex** |  |  |  | <0.001^2^ |
| Male | 13,445 (50.31%) | 13,079 (49.97%) | 366 (66.06%) |  |
| Female | 13,281 (49.69%) | 13,093 (50.03%) | 188 (33.94%) |  |
| **Age groups** |  |  |  | 0.010^2^ |
| 6-9 | 10,258 (38.38%) | 10,018 (38.28%) | 240 (43.32%) |  |
| 10-12 | 8,974 (33.58%) | 8,800 (33.62%) | 174 (31.41%) |  |
| 13-15 | 5,303 (19.84%) | 5,191 (19.83%) | 112 (20.22%) |  |
| 16-18 | 2,191 (8.20%) | 2,163 (8.26%) | 28 (5.05%) |  |
| **Height** | 145 (130, 157) | 145 (130, 157) | 140 (130, 153) | <0.001^3^ |
| **Weight** | 40 (29, 50) | 40 (29, 50) | 38 (28, 50) | 0.097^3^ |
| **Body Mass Index** | 17.9 (15.6, 21.1) | 17.9 (15.6, 21.0) | 18.1 (15.5, 21.5) | 0.426^3^ |
| **Residential area** |  |  |  | 0.414^2^ |
| City | 12,473 (46.67%) | 12,215 (46.67%) | 258 (46.57%) |  |
| City-country fringe | 6,155 (23.03%) | 6,016 (22.99%) | 139 (25.09%) |  |
| Country | 8,098 (30.30%) | 7,941 (30.34%) | 157 (28.34%) |  |
| **Annual family income (yuan)** |  |  |  | 0.197^2^ |
| Below 30,000 | 9,591 (35.89%) | 9,371 (35.81%) | 220 (39.71%) |  |
| 30,000-80,000 | 10,031 (37.53%) | 9,827 (37.55%) | 204 (36.82%) |  |
| 80,000-150,000 | 4,793 (17.93%) | 4,700 (17.96%) | 93 (16.79%) |  |
| 150,000-300,000 | 1,897 (7.10%) | 1,870 (7.15%) | 27 (4.87%) |  |
| 300,000-1000,000 | 346 (1.29%) | 337 (1.29%) | 9 (1.62%) |  |
| Above 1000,000 | 68 (0.25%) | 67 (0.26%) | 1 (0.18%) |  |
| **Primary caregiver** |  |  |  | <0.001^2^ |
| Mother | 18,425 (68.94%) | 18,077 (69.07%) | 348 (62.82%) |  |
| Father | 2,408 (9.01%) | 2,353 (8.99%) | 55 (9.93%) |  |
| Paternal grandparents | 3,601 (13.47%) | 3,499 (13.37%) | 102 (18.41%) |  |
| Maternal grandparents | 1,356 (5.07%) | 1,328 (5.07%) | 28 (5.05%) |  |
| Nanny | 21 (0.08%) | 19 (0.07%) | 2 (0.36%) |  |
| Others | 915 (3.42%) | 896 (3.42%) | 19 (3.43%) |  |
| **Primary caregiver's education** |  |  |  | 0.077^4^ |
| Junior high and below | 16,152 (60.44%) | 15,804 (60.39%) | 348 (62.82%) |  |
| Bachelor | 4,143 (15.50%) | 4,046 (15.46%) | 97 (17.51%) |  |
| Senior high | 6,314 (23.62%) | 6,207 (23.72%) | 107 (19.31%) |  |
| Master degree and above | 117 (0.44%) | 115 (0.44%) | 2 (0.36%) |  |
| **Marital status of parents** |  |  |  | <0.001^2^ |
| Non-special | 22,355 (83.65%) | 21,931 (83.80%) | 424 (76.53%) |  |
| Divorced | 2,830 (10.59%) | 2,752 (10.52%) | 78 (14.08%) |  |
| Reconstituted | 1,218 (4.56%) | 1,174 (4.49%) | 44 (7.94%) |  |
| Widowed | 323 (1.21%) | 315 (1.20%) | 8 (1.44%) |  |
| **Multiple-child family** |  |  |  | 0.971^2^ |
| No | 11,309 (42.31%) | 11,075 (42.32%) | 234 (42.24%) |  |
| Yes | 15,417 (57.69%) | 15,097 (57.68%) | 320 (57.76%) |  |
| **Gestational weeks** |  |  |  | 0.523^2^ |
| ˂32 w | 925 (3.46%) | 905 (3.46%) | 20 (3.61%) |  |
| 32–35 w | 1,244 (4.65%) | 1,216 (4.65%) | 28 (5.05%) |  |
| 35–37 w | 2,050 (7.67%) | 2,011 (7.68%) | 39 (7.04%) |  |
| Term (37–40 w) | 20,830 (77.94%) | 20,407 (77.97%) | 423 (76.35%) |  |
| ˃40 w | 1,677 (6.27%) | 1,633 (6.24%) | 44 (7.94%) |  |
| **Birth weight** |  |  |  | 0.009^4^ |
| ˂1 kg | 115 (0.43%) | 109 (0.42%) | 6 (1.08%) |  |
| 1–1.5 kg | 1,249 (4.67%) | 1,210 (4.62%) | 39 (7.04%) |  |
| 1.5–2.5 kg | 4,118 (15.41%) | 4,041 (15.44%) | 77 (13.90%) |  |
| 2.5–4 kg | 20,273 (75.85%) | 19,865 (75.90%) | 408 (73.65%) |  |
| ˃4 kg | 971 (3.63%) | 947 (3.62%) | 24 (4.33%) |  |
| **Smoking in pregnancy** | 602 (2.25%) | 587 (2.24%) | 15 (2.71%) | 0.466^2^ |
| **Alcohol drinking in pregnancy** | 384 (1.44%) | 372 (1.42%) | 12 (2.17%) | 0.145^2^ |
| **Passive smoking in pregnancy** | 3,695 (13.83%) | 3,549 (13.56%) | 146 (26.35%) | <0.001^2^ |
| **Depressed in pregnancy** |  |  |  |  |
| **mom** | 1,957 (7.32%) | 1,832 (7.00%) | 125 (22.56%) | <0.001^2^ |
| **dad** | 371 (1.39%) | 350 (1.34%) | 21 (3.79%) | <0.001^2^ |

^1^n (%); Median (IQR), ^2^Pearson's Chi-squared test, ^3^Wilcoxon rank sum test, ^4^Fisher's exact test

**Table S2: VIF Values for Variables Included in Multivariate Analysis**

| **Variables** | **VIF** |
| --- | --- |
| Sleep anxiety | 3.755 |
| Bedtime resistance | 3.552 |
| Parasomnias | 3.398 |
| Sleep disordered breathing | 2.795 |
| Night wakings | 2.369 |
| Sleep disorders | 2.361 |
| Cohesion | 2.054 |
| Conflict | 1.957 |
| Daytime sleepiness | 1.784 |
| Depression symptoms (SDS) | 1.393 |
| Sleep duration | 1.390 |
| Anxiety symptoms (SAS) | 1.293 |
| Abnormal behavior | 1.288 |
| Sleep onset delay | 1.258 |
