# Supplementary material for: Early warning signs and comorbidities of attention deficit hyperactivity disorder in children in Western China: a multicenter, cross-sectional study
Source: BMC Public Health. 2025 Dec 17;26:268. doi: 10.1186/s12889-025-26014-8 (PMC12822042; doi:10.1186/s12889-025-26014-8)
Supplement: Supplementary file 2 — Supplementary Material 2. [file 12889_2025_26014_MOESM2_ESM.pdf]

Basic Information about the Child:

1. **Child's Gender:** [Single Choice] *
○ Male ○ Female
2. **Please enter your child's age (years):** [Open-ended] *

---

3. **Current Height of the Child (cm):** [Open-ended] *
(Please provide an integer)

---

4. **Current Weight of the Child (kg):** [Open-ended] *
(Please provide an integer)

---

5. **Family's Long-term Residence:** [Single Choice] *
○ City
○ Urban-Rural Fringe
○ Rural Area
6. **Family's Economic Situation, Total Annual Income (Yuan):** [Single Choice] *
○ Below 30,000
○ 30,000 - 80,000
○ 80,000 - 150,000
○ 150,000 - 300,000
○ 300,000 - 1,000,000
○ Above 1,000,000
7. **Who Primarily Takes Care of the Child:** [Single Choice] *
○ Mother
○ Father
○ Grandparents
○ Maternal Grandparents
○ Nanny
○ Other _________________ *
8. **Educational Level of the Primary Caregiver:** [Single Choice] *
○ Junior High School or Below
○ Senior high School
○ Bachelor
○ Master's Degree or Above
9. **Current Marital Status of the Parents:** [Single Choice] *
○ Non-Special Circumstances
○ Divorced

○ Reorganized Family
○ Widowed

10. Is it a Multi-Child Family? [Single Choice] *
○ No
○ Yes

11. Gestational Age of the Child at Birth: [Single Choice] *
○ < 32 Weeks
○ 32-35 Weeks
○ 35-37 Weeks
○ Full Term (37-40 Weeks)
○ > 40 Weeks

12. Birth Weight of the Child: [Single Choice] *
○ <1 kg
○ 1-1.5 kg
○ 1.5-2.5 kg
○ 2.5-4 kg
○ >4 kg

13. Did the Mother Smoke, Drink, or Expose to Secondhand Smoke During Pregnancy? [Multiple Choices] *
□ None
□ Smoked
□ Drank Alcohol
□ Exposed to Secondhand Smoke

14. Did Either Parent Experience Emotional Distress or Depression During Pregnancy? [Single Choice] *
○ No
○ Yes, Mother
○ Yes, Father
○ Yes, Both Parents
